# Climate and pH Predict the Potential Range of the Invasive Apple Snail (*Pomacea insularum*) in the Southeastern United States

**DOI:** 10.1371/journal.pone.0056812

**Published:** 2013-02-22

**Authors:** James E. Byers, William G. McDowell, Shelley R. Dodd, Rebecca S. Haynie, Lauren M. Pintor, Susan B. Wilde

**Affiliations:** 1 Odum School of Ecology, University of Georgia, Athens, Georgia, United States of America; 2 Warnell School of Forestry and Natural Resources, University of Georgia, Athens, Georgia, United States of America; 3 School of Environment and Natural Resources, The Ohio State University, Columbus, Ohio, United States of America; Aberystwyth University, United Kingdom

## Abstract

Predicting the potential range of invasive species is essential for risk assessment, monitoring, and management, and it can also inform us about a species’ overall potential invasiveness. However, modeling the distribution of invasive species that have not reached their equilibrium distribution can be problematic for many predictive approaches. We apply the modeling approach of maximum entropy (MaxEnt) that is effective with incomplete, presence-only datasets to predict the distribution of the invasive island apple snail, *Pomacea insularum*. This freshwater snail is native to South America and has been spreading in the USA over the last decade from its initial introductions in Texas and Florida. It has now been documented throughout eight southeastern states. The snail’s extensive consumption of aquatic vegetation and ability to accumulate and transmit algal toxins through the food web heighten concerns about its spread. Our model shows that under current climate conditions the snail should remain mostly confined to the coastal plain of the southeastern USA where it is limited by minimum temperature in the coldest month and precipitation in the warmest quarter. Furthermore, low pH waters (pH <5.5) are detrimental to the snail’s survival and persistence. Of particular note are low-pH blackwater swamps, especially Okefenokee Swamp in southern Georgia (with a pH below 4 in many areas), which are predicted to preclude the snail’s establishment even though many of these areas are well matched climatically. Our results elucidate the factors that affect the regional distribution of *P. insularum*, while simultaneously presenting a spatial basis for the prediction of its future spread. Furthermore, the model for this species exemplifies that combining climatic and habitat variables is a powerful way to model distributions of invasive species.

## Introduction

Invasive species can often negatively impact native species and ecosystems, especially in cases where they spread disease or over-consume resources [1]. Early detection and prediction are central to the effective management of such invasive species to minimize negative impacts [2]. Many invasive species are still expanding their range, especially those that have recently been introduced into novel areas. Recently there has been a rise in the application of ecological niche modeling to predict habitats vulnerable to invasion, which then can guide early detection and rapid response efforts against invasive species. These modern modeling technologies not only can help predict invasions, but also identify what particular environmental factors limit a species’ distribution [3]. In many cases, these factors may be regionally correlated, which makes them conducive to utilizing data from large-scale sampling programs and even remote sensing (e.g., [4]–[6]) to model and predict sites that are vulnerable to invasion.

The *Pomacea* genus of freshwater snails is known to be quite invasive and can pose risks to agricultural crops and human and wildlife health [7], [8]. The best studied *Pomacea* species is the channeled apple snail, *P. canaliculata*, which is classified as one of the 100 “World’s Worst” invaders by the Global Invasive Species Database. It has large impacts on aquatic ecosystems as a consumer of vegetation and is among the few aquatic snail vectors of the zoonotic nematode, *rat lungworm* (*Angiostrongylus cantonensis*) [9]. A species endemic to Mexico, *Pomacea patula catemacensis,* which is a food item for humans and wildlife, was shown to accumulate appreciable levels of cyanotoxins [10]. Several invasive *Pomacea* are major agricultural pests in Asia where rice production suffered after intentional introduction as a potential food source for local consumption and export [11], [12].

**Figure 1:**
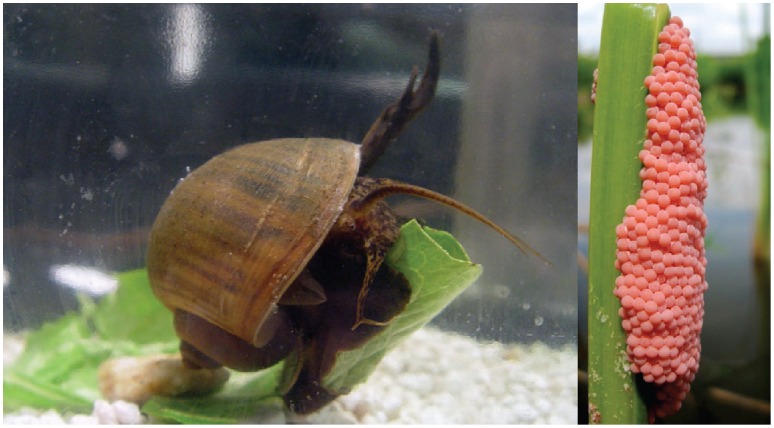
*Pomacea insularum* adult (6.1 cm) and an egg mass (7.6×2.5 cm). *P. insularum* has a channeled suture and often exceeds 10 cm in height and lays conspicuous large pink egg masses. Photo credits: (left)–Freshwater Gastropods of North America website; (right)–J. Morgan.

The island apple snail, *Pomacea insularum,* is originally native to South America and was introduced to the United States most likely in the early 1990’s, but possibly as late as 2002 [7] (Figure 1). Since then, it has rapidly spread from its initial introduced populations in Texas and Florida, and has been documented throughout eight southeastern states in the USA [7], [13], [14]. The limited ecological data on *P. insularum* in the USA show that the species has considerable impacts, especially on native aquatic vegetation and snail species [15]–[17]. In Florida, in particular, *P. insularum* is much larger and more fecund than the native *P. paludosa* (e.g., *P. insularum* egg clutches contain 2000 eggs as opposed to its native counterpart which produces 20–30 eggs) [18]–[20]. The invasion of *P. insularum* has possibly affected the endangered snail kite, a specialist predator on the native *P. paludosa*, which seemingly experienced decreased foraging success and juvenile survival following invasion of *P. insularum* [21]. Thus, predicting habitats that are vulnerable to further invasion by *P. insularum* is of particular conservation interest from the perspective of preventing species replacement and larger ecosystem impacts.

Our objectives were to build a predictive model of *P. insularum* distribution in the southeastern USA and identify which environmental variables best predict its distribution using Maximum Entropy modeling (MaxEnt). MaxEnt models use geo-referenced species occurrence records and data on environmental variables to generate a predictive continuous probability distribution of habitat suitability over a spatial domain. MaxEnt is effective with incomplete, presence-only datasets and is a useful approach when modeling a species not at equilibrium, or one that has detection issues [22]. MaxEnt has been shown to consistently outperform more established methods of estimating species’ distributions from occurrence data (e.g., Genetic Algorithm for Rule Set Production- GARP, BIOCLIM models) and has been increasingly applied to predicting the distribution of invasive species in both terrestrial and aquatic systems [23]–[25]). MaxEnt models have been increasingly utilized, especially with advances in the collection of large scale environmental data (e.g., [26], [27]).

Our MaxEnt model uses bioclimatic data to predict the distribution of *P. insularum* in the southeastern USA. Although the large scale climate modeling provides an excellent initial estimate of the snail’s abiotic tolerances, we also discuss known tolerances of the snail to habitat and non-climate related environmental factors that can provide further guidance for refining which water bodies within the proper climate zone may be inhospitable to invasion. In particular, we focus on pH as a secondarily influential variable and layer this variable onto the model to refine the snail’s predicted conceivable distribution.

## Methods

### Model Areas of Current and Predicted Climatic Suitability for *P. insularum* in the USA

We collected *P. insularum* distribution data from the USGS Non-indigenous Aquatic Species database [14]. In spring and summer of 2011 and 2012 we surveyed and spot-checked many of these sites, especially in Georgia (including two newly reported sites), to verify the presence of *P. insularum* (Table S1). Because these sites are on the advancing edge of the species’ distribution, their inclusion heavily influences the model. Also, since habitat at the edges of the snail’s distribution may have been colonized more recently or may not be as suitable, we needed to ascertain if the populations showed evidence of persistence.

*P. insularum* individuals can be difficult to differentiate morphologically from *P. canaliculata*. We relied on the findings of Rawlings et al. (2007) whose genetic work indicated that *P. canaliculata* was mostly restricted to Arizona and California locations, while all *Pomacea* populations in Georgia were *P. insularum.* Furthermore, the field surveyors (R. Haynie and S. Robertson) were familiar with the appearance of *P. insularum* egg masses, which are strikingly different to a trained observer, than those of *P. canaliculata.* Nonetheless, we tested the robustness of our model by performing a sensitivity analysis (Materials S1), and found our model was extremely robust to the potential of misidentified populations. Because *P. insularum* is an invertebrate and none were collected, no specific permits were required for the described field studies. We accessed most sites through public boat landings or publicly owned right-of-ways. However, three of the sites required permission to access; one at Alma, Georgia by a private landowner (Charles Douglas) and two at St. Marys by the city (Chris Cox, St. Marys Dept of Public Works).

We gathered environmental data for climate variables from WorldClim to use as predictive variables in the MaxEnt modeling of *P. insularum*’s distribution. In particular, we began by using 19 bioclimatic variables (i.e. BioClim variables), which were derived from monthly temperature and rainfall values (Table 1). Colinearity of predictor variables in MaxEnt models can lead to spurious results [28]–[30], and BioClim variables are often collinear. Thus, we calculated the Pearson’s correlation coefficient of all combinations of the 19 BioClim variables for known point locations of *P. insularum*. Variables were then selected *a priori* for their biological importance in affecting apple snail populations and to minimize the number of correlation coefficients above 0.6. Using these criteria, two temperature variables [maximum temperature of the warmest month (Bio5) and minimum temperature in the coldest month (Bio6)] and three precipitation variables [annual precipitation (Bio12), precipitation of the driest quarter (Bio17), and precipitation of the warmest quarter (Bio18)] were selected as predictor variables. These variables were chosen over others with which they correlated strongly because *P. insularum* is known to experience mortality at low temperatures [31], and fluctuations in water levels can have impacts on snail reproduction and juvenile survival [32]. A smoothing function (a regularization multiplier of ten) was used to smooth response curves of variables to prevent overfitting of the response curve for each variable.

**Table 1:** List of environmental variables from the BIOCLIM dataset used in the MaxEnt model.

BIOCLIM predictor variables	
1. annual mean temperature	10. mean temperature of warmest quarter
2. mean diurnal range temperature	11. mean temperature of coldest quarter
3. isothermality	**12. annual precipitation**
4. temperature seasonality	13. precipitation of wettest month
**5. maximum temperature of warmest month**	14. precipitation of driest month
**6. minimum temperature of coldest month** †	15. precipitation seasonality
7. temperature annual range	16. precipitation of wettest quarter
8. mean temperature of wettest quarter	**17. precipitation of driest quarter**
9. mean temperature of driest quarter	**18. precipitation of warmest quarter** †
	19. precipitation of coldest quarter

Bold font indicates variables considered in initial model run;

† superscript indicates the two variables included in final model.

Because *P. insularum* records and sampling efforts are focused on the Gulf Coast and Southeastern regions of the USA, we limited the scope of our model to the southeastern USA (Florida, Georgia, Alabama, Mississippi, Louisiana, Texas, South Carolina, North Carolina, and Virginia). To avoid sampling bias (overweighing the model) from closely located points in heavily studied areas, point locations were converted into a presence/absence grid with the same scale (1×1 km) as the predictor BioClim variables, and then converted back to a single point from the center of each grid where *P. insularum* was present. The final list of presence points for *P. insularum* used in our model are presented in Table S2.

MaxEnt generates a continuous probability surface of habitat suitability by comparing values of environmental variables (e.g., climate) at known species’ locations to values at other locations (e.g., background points) throughout the area of inference. We limited the distribution of background points to the southeastern states listed above. Background points were created by randomly placing 10,000 points across our focal area.

We then used MaxEnt version 3.3.3e to generate a model of *P. insularum* distribution using the five climatic predictor variables [33]. A total of ten runs of the model were completed, with a random 10% of the 68 known locations sampled without replacement and set aside for model validation for each model run. If any of the five predictor variables did not contribute significantly to the model, as measured by an overall importance score of 5% or less, they were removed from the model and the more parsimonious model was re-run. We determined the relative importance of variables remaining in the model with permutation of importance. Permutation of importance is a measure of the contribution of each variable quantified by the resulting decrease in training AUC when randomly permuting a variable. The values total to 100% across all variables and larger decreases mean the model depends more heavily on that variable.

An important step in evaluating the model is to verify that the data used to train and test the model performed significantly better than random. A typical approach is to look at the area under the curve (AUC) score associated with the models. An AUC score of 0.5 indicates a model is no better than random while 1.0 indicates the model distinguishes perfectly between presence and absence of a species. However, there are criticisms of reporting an AUC score by itself, particularly for a species that is not at equilibrium [34], [35]. To address this, we also report a graph of the receiver operating curve (ROC). The logistic output from MaxEnt, is scaled between 0–1; however, interpretation is made easier in most cases by defining thresholds of habitat suitability. To establish which areas are climatically suitable for *P. insularum*, we used two different commonly used thresholds based on the average model outputs. One, the “correctly classify all points” threshold, uses looser criteria so that it would correctly classify all known *P. insularum* points. By correctly classifying all known *P. insularum* points, a larger area will be designated as suitable habitat compared to other thresholds. A second, stricter, threshold uses tighter criteria that allows up to 10% of known *P. insularum* points to be misclassified. This has the effect of conservatively identifying a region of highest fit that does not allow outlying points of *P. insularum* presence to expand the predicted area of occupancy beyond a core region.

### Enhancing Predictions of Apple Snail Distribution with Non-climatic Variables

To identify the tolerance range of *P. insularum* to different abiotic variables and thus refine the predictions of the snail’s distribution based solely on climate variables, we conducted a literature review of both published and unpublished studies (e.g. reports, websites) focusing on known influential water properties: salinity, pH, and temperature, as well as emersion and desiccation tolerances [31] (Table 2). We chose to focus on pH, since pH and hardness may be limiting factors for growth (especially shell maintenance) and hatchling success in nonindigenous *Pomacea sp*. [31], [36]. Also, low pH in general is known to preclude shell construction in many molluscs, including apple snails [37]–[40]. Furthermore, pH is a tracked variable with a large amount of variability throughout the southeastern USA.

**Table 2:** Experimentally determined incipient physiological tolerance limits under laboratory conditions for adult and juvenile *Pomacea insularum* collected in Texas (from Ramakrishnan [31]).

Physiological parameter	Lower limit	Upper limit
Salinity	0.0‰	6.8–10.2‰
pH	3.5–4.0	10–10.5
Temperature	15.23°C	36.6°C
Emersion	70 days at 30°C, <5% relative humidity	>308 days at 20–25°C, >75% relative humidity

For salinity and pH the ranges of values bracket the median lethal values at 28 days exposure (LD_50_/28). Temperature limits were statistically calculated from experimental data to yield the temperatures at which 99% mortality occurred in 28 days (LTp_99_). Emersion values are the maximum observed survival time of the snail out of water at the stated temperature and humidity.

To examine how pH might affect snail distribution, we downloaded pH measurements from the EPA STORET database, which contains surface water quality data for the entire USA [41]. In the case of Florida, only the last five years of data were used due to the large number of records in the state, and limits on the maximum number of records in a STORET search. For sites with multiple measurements, the lowest recorded measurement was used, because low pH can lead to mortality in *P. insularum* in fewer than 10 days [31]. We investigated further any locations with a pH below 2 since such a value is suspiciously low for a natural water body. If other measurements at that location showed substantially higher pH, we used the second lowest pH recorded for the location. These points were then used to create a continuous map of pH distributions in the southeastern United States using Kriging, a spatial analysis approach used to interpolate between points. A similar approach has been used for demonstrating regional patterns in the pH of lakes [42], including one study examining the potential for zebra mussel expansion [43]. In total, the pH data layer was generated from point measurements of pH at 35,000 different locations in the southeast.

On top of the MaxEnt map predicting the influence of climate variables, we plotted areas representing two different pH thresholds. One threshold was a pH <4 as determined by Ramakrishnan [31] to be lethal for *P. insularum* (Table 2). A second, less extreme threshold was a pH = 5.5, which was determined by Bernatis (unpublished data) to be lethal to *P. insularum* hatchlings (median lethal dose over 3 days, or LD_50_/3). Although these pH layers could have been formally included in the maximum entropy modeling, due to the high amount of spatial variability in pH measurements, we felt more comfortable viewing the interpolated pH layer as a rough guide to pH values in an area. Specifically, the pH layer is good at reflecting large scale regional processes that drive low pH, such as underlying soil and bedrock composition and organic matter inputs. However, it is not as good at reflecting small scale variation that is unlikely to be driven by regionally correlated processes. For example, because of its size and position within the landscape, a roadside mud puddle would probably not be affected by the larger scale drivers of low pH like a big swamp in a region would.

## Results

### Maximum Entropy Modeling

Three point locations were discarded from the USGS invasive species database due to our verification process. First, we were unable to verify the Savannah River (Cowden, SC) population during two separate field surveys. The initial report, of numerous shells (but no live snails or eggs) was made by a U.S. Fish and Wildlife Service malacologist and therefore had been considered highly reliable (L. Zimmerman, pers comm). We did document both live and dead Japanese mystery snails, *Bellamya japonica,* (or possibly Chinese mystery snails, *B. chinensis*), at this site during our April 2012 survey. Second, the point location in Fort Worth, Texas was removed from the dataset because the population had apparently been extirpated [13], [44], [45]. In both these cases the initial sightings seem highly reliable and we presume that cold winter temperatures may not have allowed the populations to persist. A third point in Arizona was eliminated because a recent genetic study identified that population of apple snails as a different *Pomacea* species [7].

**Figure 2:**
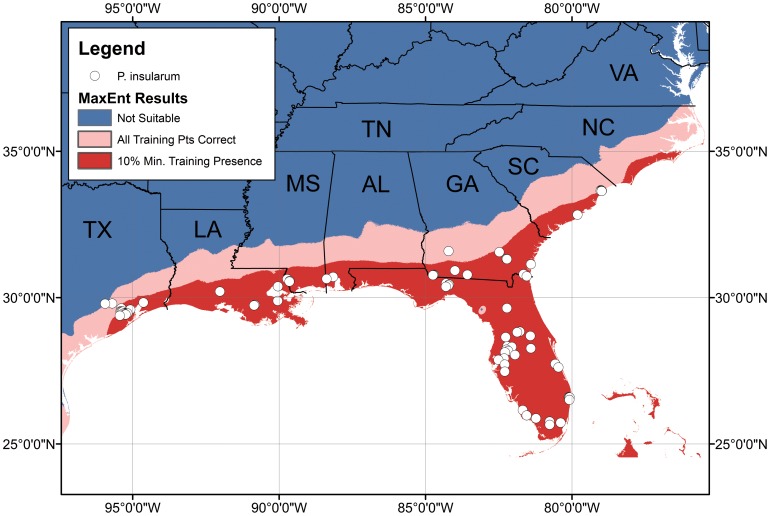
Present populations of the island apple snail, *Pomacea insularum,* and its occupiable area. Map shows the southeastern United States. As predicted by the maximum entropy model, red represents areas with the highest climatic compatibility for the snail as determined by using an inclusion threshold that correctly classifies all sites above the minimum 10% training omission threshold. Pink represents areas determined to be suitable by using the less stringent threshold calculated by correctly classifying all known *P. insularum* points above the minimum training presence.

**Figure 3:**
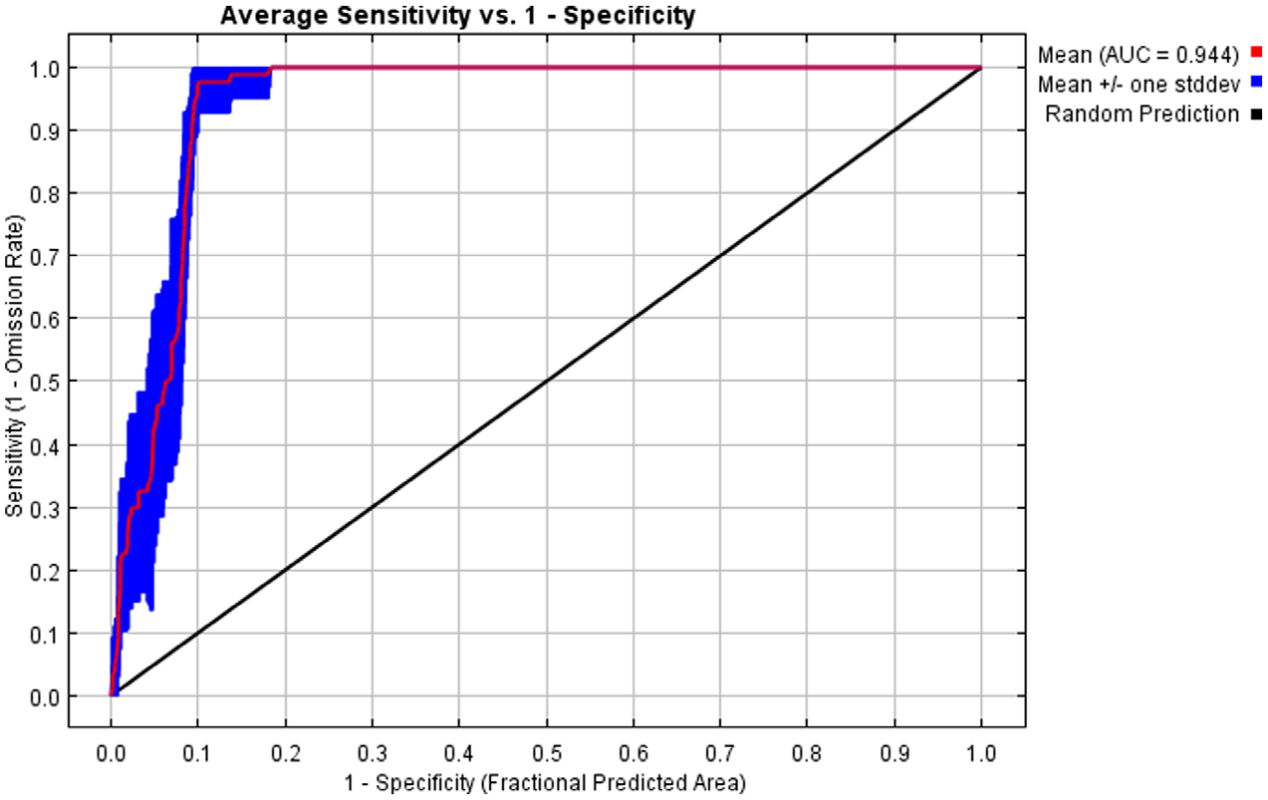
The average receiver operating curve from the ten model runs showing relative specificity and sensitivity. One standard deviation above and below the average curve is shown in blue. Area Under the Curve (AUC) is calculated from this curve.

Only two of our initial five climatic variables were selected as significant contributors to the distribution model of *P. insularum*: minimum temperature in the coldest month (Bio6) and precipitation in the warmest quarter (Bio18). Minimum temperature in the coldest month was the more important variable of the two, with a 60.6% permutation of importance. More precipitation in the warmest quarter and higher minimum temperatures were associated with an increased likelihood of the climate being suitable for *P. insularum*. From the ten model runs, the average AUC was 0.94, with little variation in AUC between runs. The averaged output from these ten model runs is shown in Figure 2. The good fit of the model is furthered evidenced by the average receiver operating curve (Figure 3). All of Florida, as well as coastal Louisiana, Alabama, Mississippi, Georgia, South Carolina, and portions of coastal Texas and North Carolina were reflected to have the highest climatic compatibility as determined by using an inclusion threshold that correctly classifies all sites above the minimum 10% training omission threshold. Coastal regions in pink represent areas determined to be suitable by using the less stringent threshold calculated by correctly classifying all known *P. insularum* points above the minimum training presence. Areas farther inland in the previously mentioned states, were classified as suitable using this less restrictive inclusion threshold that correctly classifies all known *P. insularum* points.

### pH as an Additional Factor

**Figure 4:**
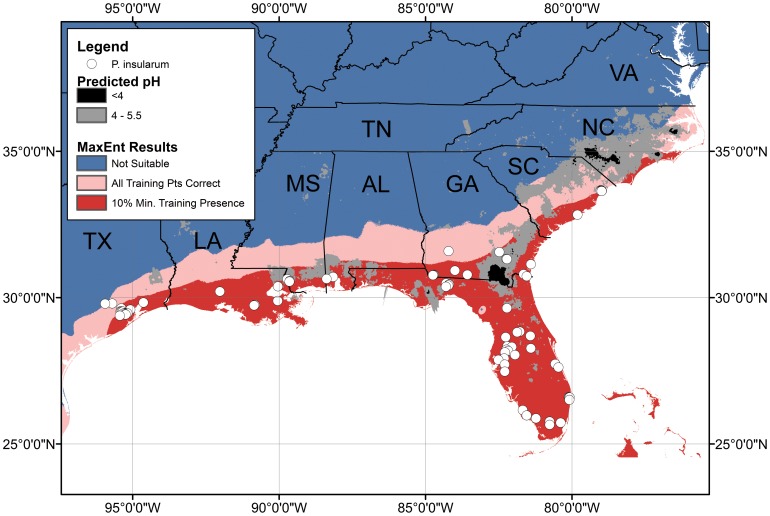
Map of predicted *P. insularum* distribution also showing areas with low pH values. Acidic waters may inhibit the invasion of *P. insularum*. Black represents areas with a predicted pH <4, the mortality threshold determined by Ramakrishnan [31] and gray areas have a predicted pH of 4–5.5, below the hatchling mortality threshold determined by Bernatis (unpublished data). The large black area in southern Georgia is Okefenokee Swamp discussed in the text.

Areas with inappropriate pH for apple snails are fairly abundant throughout a large swath of otherwise likely areas of establishment for *P. insularum* (Figure 4). Of areas predicted to be in highly favorable climatic zones, the Okefenokee Swamp in southern Georgia appears to have a low enough pH to strongly prohibit *P. insularum* from invading (Figure 4, Table 2). The majority of the coastal plain of North Carolina appears inhospitable due to the combination of large areas with low pH coupled with a limited area of favorable climate.

## Discussion

*Pomacea insularum* has spread rapidly in the southeastern USA only during the last decade. Our maximum entropy model indicates that the coastal plain from Texas to South Carolina is at high risk for *P. insularum* invasion based on climate suitability. The coastal plain of North Carolina appears less at risk because of both borderline climate conditions and widespread low pH (Figure 4). Knowledge of the necessary physical environmental attributes for invasion aids greatly in understanding issues regarding *P. insularum*’s regional and microscale distribution, as well as the efficacy of possible control measures via alteration of physical variables. Identifying the regions where climatic conditions are suitable for *P. insularum* informs managers of the risks and directs regional efforts to monitor water bodies for early signs of an invasion. Because *P. insularum* can be easily identified through its bright pink egg masses laid above the waterline, early detection is possible.

Our results indicate that the minimum temperature in the coldest months and maximum amount of precipitation in the warmest months are the best predictors of the nineteen variables included in the BioClim database. For shallow or smaller water bodies, these climatic variables equate to warmer overwintering temperatures and more permanent aquatic habitat (i.e. reduced chance of desiccation). These abiotic variables should directly reflect the likelihood of surviving freezing and desiccation. However, they may interact with important biological variables, like predation, which should also affect invasion success. Fecundity and survival of *P. insularum* has been found to be negatively associated with habitat permanence because of increased abundance and diversity of predators in more permanent water bodies [46]. This may suggest that the habitats most vulnerable to high impacts (e.g. via high densities of apple snails) are those with an intermediate level of habitat permanence (which might explain the important, but less influential role of precipitation of the two significant climate variables).

Known locations with *P. insularum* exhibit average minimum monthly temperatures as low as 6°C (Charleston, SC), and uninvaded areas modeled as suitable have minimum monthly temperatures as low as 2°C (Wilmington, NC). These values nicely bracket the average minimum monthly temperatures of 4–6°C observed in Buenos Aires near the colder extreme of the snail’s native range [7], [12]. Ramakrishnan [31] had demonstrated a lethal minimum temperature of 15°C in the laboratory (Table 2), however in those trials the temperature was held low for 28 consecutive days. Differences in minimum monthly temperatures between studies might be a consequence of the duration of exposure to low temperatures. For example, the thresholds for low temperature tolerance over a month-long period may be substantially higher than tolerance of low temperature events of shorter duration. It is also possible that the snails have behavioral mechanisms to tolerate low temperatures, such as burrowing, which could not be exhibited in laboratory experiments [8].

The BioClim temperature variables themselves stem from air temperatures. These values have been used in successful models of aquatic species [25], [47]–[49] because the values typically correlate well with water temperatures. However, in some cases the tight correlation between air and water temperatures may break down, particularly in fast moving water or deep water bodies. Given that *P. insularum* commonly colonizes small water bodies, such as roadside ditches and littoral edges of larger water bodies, the air temperatures should correlate well with temperatures of its favored aquatic environments.

Although the climate model predicts that southern Georgia is a highly suitable area for *P. insularum*, low pH values may exclude the snail from many areas such as the Okefenokee Swamp and other black water swamps of the southeastern coastal plain. The acidic waters appear to limit the snail’s spread in an otherwise hospitable climate. There are several important caveats to our approach. It is important to recognize that the Kriging used to create the predicted pH layer smoothes out the extreme points and only provides a general guideline for the expected pH values for water bodies in the region. There can be significant departures from the predicted pH for individual water bodies, particularly for smaller water bodies and ones that are man-made. In fact, *P. insularum* can thrive in very small water bodies such as roadside ditches [13], [50]. Any bodies of water that depart from the expected pH within the appropriate climate zone may be vulnerable to invasion, and a local assessment of pH in an area would greatly benefit any management plan and monitoring. As a case in point, two populations of island apple snails are found in southern Georgia within the area that has a minimum predicted pH of below 5.5 based on STORET measurements. One of these water bodies was an isolated pond in the flood plain of the Alabaha River and the other was in the main stem of the river. However, in the sites where these populations were found, a quarter of STORET pH values were >5.5 and our own measurements at the time of snail surveys had a pH >7. As with any measurement where temporal variation is involved, care should be taken in interpretation, especially in areas where the minimum predicted pH is near the tolerance threshold. Thus, while low pH may make an overall region *on average* more resistant to *P. insularum* establishment, in localized areas, *P. insularum* may still be able to establish.

The spread of *P. insularum* is concerning because of at least two particularly worrisome ecological impacts. First, *P. insularum* voraciously consumes aquatic vegetation. Together with invasions of the closely related congener, *P. canaliculata*, their high consumption of vegetation has led to a regime shift from aquatic plants to a system dominated by algae, and often cyanobacteria [51]. Many natural wetlands surveyed in Thailand showed a strong association of high densities of the snail with almost complete absence of aquatic plants. Second, *Pomacea* sp. can bioaccumulate algal toxins, which poses a threat to a variety of organisms that feed on both the native and invasive snails [10]. Recent laboratory studies have demonstrated that *P. insularum* can transfer the neurotoxin linked to Avian Vacuolar Myelinopathy (AVM) to its avian predators [52]. This often lethal neurologic disease affects waterbirds and their avian predators in the Southern United States and has been linked to a novel cyanobacterium that grows as an epiphyte on submerged aquatic vegetation [53], [54] including the invasive *Hydrilla verticillata*, a preferred food source for *P. insularum* [17]. The presence of the invasive apple snails may substantially increase the risk of AVM for local bird populations, particularly the molluscivorous, federally-listed endangered Everglade snail kite (*Rostrhamus sociabilis plumbeus*) [52]. There is a further, but largely unexplored risk that *P. insularum* harbors rat lungworm parasite (*Angiostrongylus cantonensis*), a nematode that causes eosinophilic meningitis in humans and has been reported in invasive populations of *P. canaliculata* in Asia [9].

Our results provide insight into the factors that affect the distribution of *P. insularum* and present a spatial basis for the prediction of its future spread. Many niche models are based purely on climate variables because these data are readily available, covering large spatial scales. When possible, adding in other data layers can greatly enhance predictions (e.g., [48], [55], [56]). An additional advantage of the maximum entropy approach is that it is effective with incomplete, presence-only datasets [22]. The thoroughness of sampling is always an issue to consider with any species distribution modeling effort, but especially for an invasive species whose distribution is not likely yet at equilibrium. Furthermore, the habitat suitability map that results from modeling with this approach can be an essential visualization tool to galvanize action by resource managers on potentially harmful species [57]. Predicting the spread and potential range of non-native species helps prioritize areas for vigilance and management, while also informing a species’ overall potential invasiveness.

## Supporting Information

Table S1 Descriptions and locations of field sites surveyed in 2011.
(DOCX)

Table S2 List of all *Pomacea insularum* presence sites used in MaxEnt model.
(DOCX)

Materials S1 **Information and test supporting robustness of MaxEnt model predictions.**
(DOCX)
