# Supplementary material for: Climate and pH Predict the Potential Range of the Invasive Apple Snail (Pomacea insularum) in the Southeastern United States
Source: PLoS One. 2013 Feb 22;8(2):e56812. doi: 10.1371/journal.pone.0056812 (PMC3579942; doi:10.1371/journal.pone.0056812)
Supplement: Table S1 — Descriptions and locations of field sites surveyed in 2011. (DOCX) [file pone.0056812.s001.docx]

Table S1: Sites we surveyed in 2011 based on previously reported *P. insularum* sightings. The superscripts on sites denote from where we received the initial report of *P. insularum* presence. All counties surveyed are within the state of Georgia except for the listing for Cowden, South Carolina. The Cowden, SC entry in bold was the only population that was not confirmed. It appears to have been an ephemeral population.

| **Site** | **Subsite** | **Lat/Long** | **County** | **Drainage** | **Year**  **Reported** |
| --- | --- | --- | --- | --- | --- |
| St. Simons^1^ | Golf course pond | 31.150783  -81.401633 | Glynn | Cumberland | 2005 |
| St. Mary’s^1^ | Durango Mill Pond | 30.733467  -81.545333 | Camden | St. Mary’s | 2007 |
| St. Mary’s^1^ | Aquatic Center Pond | 30.73881  -81.55098 | Camden | St. Mary’s | 2007 |
| Blackshear^1^ | Ponds off Hwy 84 adjacent to Alabaha River | 31.315233  -82.225583 | Pierce | Satilla | 2005 |
| Alma^1^ | Lake Lure | 31.553217  -82.482350 | Bacon | Satilla | 2007 |
| **Cowden, SC^1^** | **Adjacent to Savannah River** | **33.275983**  **-81.833583** | **Aiken, SC** | **Middle Savannah** | **2008** |
| Quitman^1^ | Sunset Lake | 30.780733  -83.5734 | Brooks | Withlacoochee | 2007 |
| Lake Seminole^2^ | Flint River Arm | 30.76604  -84.75134 | Decatur | Flint | 2008 |
| Albany^3^ | Retention pond, canal | 31.591327  -84.21703 | Dougherty | Flint | 2010 |
| Thomasville^3^ | Ochlockonee River mainstem | 30.9244  -83.99967 | Thomas | Ochlockonee | 2010 |

^1^([Benson 2010](#_ENREF_3)), ^2^Jack Wingate, ^3^Dean Barber (Georgia DNR), personal communication

References:

Benson AJ (2010) *Pomacea insularum*. USGS Nonindigenous Aquatic Species Database, Gainesville, FL. Revision Date: 10/8/2009 ed.
