# Supplementary material for: Climate and pH Predict the Potential Range of the Invasive Apple Snail (Pomacea insularum) in the Southeastern United States
Source: PLoS One. 2013 Feb 22;8(2):e56812. doi: 10.1371/journal.pone.0056812 (PMC3579942; doi:10.1371/journal.pone.0056812)
Supplement: Table S2 — List of all Pomacea insularum presence sites used in MaxEnt model. (DOCX) [file pone.0056812.s002.docx]

Table S2. All presence sites used in MaxEnt model. These coordinates were converted from points, to a raster grid with the same scale as the BioClim data, then back to points in order to remove multiple points in the same BioClim grid square (1x1 km). Source: USGS Nonindigenous Aquatic Species Database and field sampling in Georgia.

| Point | latitude | longitude |
| --- | --- | --- |
| 1 | 33.67288 | -79.0146 |
| 2 | 33.66455 | -79.0146 |
| 3 | 33.64788 | -79.0229 |
| 4 | 33.64788 | -78.9896 |
| 5 | 33.64788 | -78.9813 |
| 6 | 33.63955 | -78.9896 |
| 7 | 32.83122 | -79.8146 |
| 8 | 31.58955 | -84.2146 |
| 9 | 31.55622 | -82.4813 |
| 10 | 31.32288 | -82.2396 |
| 11 | 31.31455 | -82.2229 |
| 12 | 31.13955 | -81.4063 |
| 13 | 30.92288 | -83.9979 |
| 14 | 30.79788 | -81.6563 |
| 15 | 30.78122 | -84.7146 |
| 16 | 30.78122 | -83.5729 |
| 17 | 30.77288 | -84.7396 |
| 18 | 30.77288 | -84.7313 |
| 19 | 30.76455 | -84.7479 |
| 20 | 30.73122 | -81.5479 |
| 21 | 30.70622 | -88.1563 |
| 22 | 30.63955 | -88.3813 |
| 23 | 30.63122 | -89.7229 |
| 24 | 30.56455 | -89.6396 |
| 25 | 30.54788 | -89.6479 |
| 26 | 30.48955 | -84.2979 |
| 27 | 30.44788 | -84.1979 |
| 28 | 30.41455 | -84.2229 |
| 29 | 30.38122 | -90.0396 |
| 30 | 30.37288 | -84.3063 |
| 31 | 30.20622 | -92.0229 |
| 32 | 29.88955 | -90.0479 |
| 33 | 29.83122 | -94.6313 |
| 34 | 29.79788 | -95.6979 |
| 35 | 29.78955 | -95.9396 |
| 36 | 29.74788 | -90.8313 |
| 37 | 29.73122 | -90.8563 |
| 38 | 29.63955 | -82.2229 |
| 39 | 29.63122 | -95.3896 |
| 40 | 29.58955 | -95.0813 |
| 41 | 29.53122 | -95.4146 |
| 42 | 29.53122 | -95.3896 |
| 43 | 29.52288 | -95.3646 |
| 44 | 29.52288 | -95.3313 |
| 45 | 29.47288 | -95.2563 |
| 46 | 29.47288 | -95.2479 |
| 47 | 29.46455 | -95.2313 |
| 48 | 29.46455 | -95.2229 |
| 49 | 29.46455 | -95.2063 |
| 50 | 29.46455 | -95.1813 |
| 51 | 29.45622 | -95.3146 |
| 52 | 29.43955 | -95.2646 |
| 53 | 29.42288 | -95.2479 |
| 54 | 29.42288 | -95.2396 |
| 55 | 29.38122 | -95.4396 |
| 56 | 28.83955 | -81.7813 |
| 57 | 28.81455 | -81.8729 |
| 58 | 28.68955 | -81.4229 |
| 59 | 28.64788 | -82.2646 |
| 60 | 28.33955 | -82.2063 |
| 61 | 28.31455 | -82.0563 |
| 62 | 28.26455 | -81.4063 |
| 63 | 28.22288 | -82.2396 |
| 64 | 28.18122 | -82.1813 |
| 65 | 28.13122 | -82.2896 |
| 66 | 28.03955 | -81.9479 |
| 67 | 27.92288 | -82.3146 |
| 68 | 27.87288 | -82.5146 |
| 69 | 27.73122 | -80.5729 |
| 70 | 27.71455 | -82.2979 |
| 71 | 27.61455 | -80.4646 |
| 72 | 27.47288 | -82.2979 |
| 73 | 26.59788 | -80.1146 |
| 74 | 26.58955 | -80.0979 |
| 75 | 26.58955 | -80.0813 |
| 76 | 26.50622 | -80.0896 |
| 77 | 26.15622 | -81.6813 |
| 78 | 25.97288 | -81.5479 |
| 79 | 25.87288 | -81.2313 |
| 80 | 25.76455 | -80.7646 |
| 81 | 25.72288 | -80.3896 |
| 82 | 25.65622 | -80.7646 |
