# Supplementary material for: Climate and pH Predict the Potential Range of the Invasive Apple Snail (Pomacea insularum) in the Southeastern United States
Source: PLoS One. 2013 Feb 22;8(2):e56812. doi: 10.1371/journal.pone.0056812 (PMC3579942; doi:10.1371/journal.pone.0056812)
Supplement: Materials S1 — Information and test supporting robustness of MaxEnt model predictions. (DOCX) [file pone.0056812.s003.docx]

**Supplemental Materials S1**. Testing robustness of our model predictions to misidentification of *Pomacea insularum* and its morphologically similar congener *P. canaliculata*.

Rawlings et al. (2007) states that *Pomacea canaliculata* occurred only in California and Arizona and *P. insularum* occurred in Texas, Georgia, and Florida, but they suggest in their conclusions that a population of *P. canaliculata* is possibly present in northern Florida. To be clear, the USGS database has many points that come from Rawlings et al. (2007) who confirmed many Florida populations of *P. insularum* with genetic evidence. Furthermore, because *P.* *canaliculata* and *P. insularum* are closely related congeners (Hayes et al. 2008) that could be deemed ecological equivalents, the physical parameters that will affect both snails are probably similar, if not identical. So, in theory, the model should be robust to mis-identifications for such a similar species.

Nevertheless, to test the proposition that our model is robust to any misidentifications, we performed a sensitivity analysis. For this test we created the most extreme scenario possible—that every *Pomacea* population in Florida is incorrectly identified such that there are no actual *P. insularum* populations in the whole state. Again, we know this scenario is certainly not the case given the work by Rawlings et al. (2007) that genetically verified many *P. insularum* populations in Florida. However, we wanted to try this extreme approach as a demonstration of our model’s robustness. With all snail populations removed from Florida, our MaxEnt model, still produces a nearly identical prediction for *P. insularum*’s distribution as the one in our manuscript, including incorporating almost the entirety of Florida (Figure A, below).


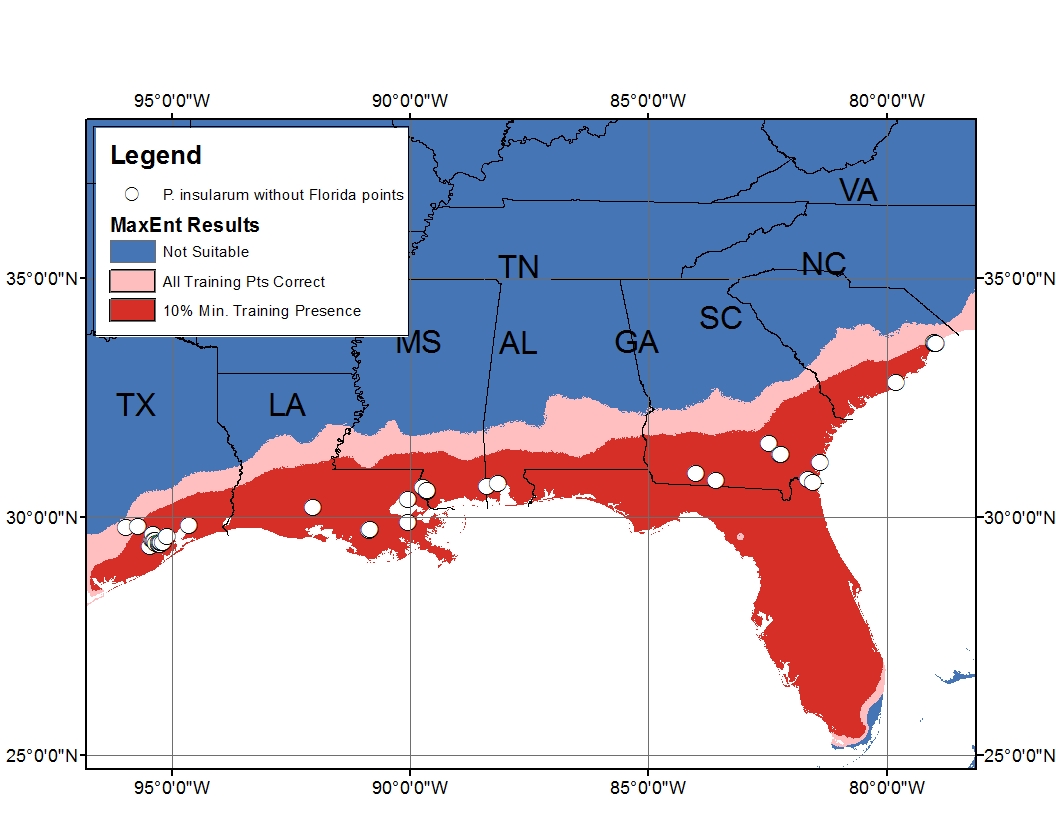


Fig. A. MaxEnt model of predicted *P. insularum* distribution with all Florida populations removed from model input.

References:

Hayes KA, Joshi RC, Thiengo SC, Cowie RH (2008) Out of South America: multiple origins of non-native apple snails in Asia. Diversity and Distributions 14: 701-712.

Rawlings TA, Hayes KA, Cowie RH, Collins TM (2007) The identity, distribution, and impacts of non-native apple snails in the continental United States. BMC Evolutionary Biology 7: Doi 10.1186/1471-2148-1187-1197.
